# Optimizing the use of low-frequency ultrasound for bacterial detachment of in vivo biofilms in dental research—a methodological study

**DOI:** 10.1007/s00784-023-05397-1

**Published:** 2023-12-23

**Authors:** Cassandra Rux, Annette Wittmer, Anja Stork, Kirstin Vach, Elmar Hellwig, Fabian Cieplik, Ali Al-Ahmad

**Affiliations:** 1 Department of Operative Dentistry and Periodontology, Medical Center, Faculty of Medicine, University of Freiburg, Hugstetter Str. 55, 79106 Freiburg, Germany; 2 Institute of Medical Microbiology and Hygiene, Faculty of Medicine, University of Freiburg, Hermann-Herder- Str. 11, 79104 Freiburg, Germany; 3 Institute for Medical Biometry and Statistics, Faculty of Medicine, University of Freiburg, Stefan-Meier-Str. 26, 79104 Freiburg, Germany; 4 Department of Conservative Dentistry and Periodontology, University Hospital Regensburg, Franz-Josef-Strauß-Allee 11, 93053 Regensburg, Germany

**Keywords:** Low-frequency ultrasound, Biofilm removal, Desorption, Sonication, Initial biofilm, Bovine enamel slabs

## Abstract

**Objectives:**

Low-frequency, low-intensity ultrasound is commonly utilized in various dental research fields to remove biofilms from surfaces, but no clear recommendation exists in dental studies so far. Therefore, this study aims to optimize the sonication procedure for the dental field to efficiently detach bacteria while preserving viability.

**Materials and methods:**

Initial biofilm was formed in vivo on bovine enamel slabs (*n* = 6) which were worn by four healthy participants for 4 h and 24 h. The enamel slabs covered with biofilm were then ultrasonicated ex vivo for various time periods (0, 1, 2, 4, 6 min). Colony-forming units were determined for quantification, and bacteria were identified using MALDI-TOF. Scanning electron microscopic images were taken to also examine the efficiency of ultrasonications for different time periods.

**Results:**

Ultrasonication for 1 min resulted in the highest bacterial counts, with at least 4.5-fold number compared to the non-sonicated control (*p* < 0.05). Most bacteria were detached within the first 2 min of sonication, but there were still bacteria detached afterwards, although significantly fewer (*p* < 0.0001). The highest bacterial diversity was observed after 1 and 2 min of sonication (*p* < 0.03). Longer sonication periods negatively affected bacterial counts of anaerobes, Gram-negative bacteria, and bacilli. Scanning electron microscopic images demonstrated the ability of ultrasound to desorb microorganisms, as well as revealing cell damage and remaining bacteria.

**Conclusions:**

With the use of low-frequency, low-intensity ultrasound, significantly higher bacterial counts and diversity can be reached. A shorter sonication time of 1 min shows the best results overall.

**Clinical relevance:**

This standardization is recommended to study initial oral biofilms aged up to 24 h to maximize the outcome of experiments and lead to better comparability of studies.

## Introduction

Biofilms are widely spread and pose tremendous challenges in various fields, including the food industry, water management, medicine, and dentistry due to their unique properties. In dentistry, they are involved in the pathogenesis of caries and periodontal diseases, as well as peri-implantitis [1, 2]. Biofilms are also the subject of numerous studies in dental material research [3–5]. In medicine, biofilm infections are the second most common cause of prosthetic joint failure [6]. With the increasing use of implants in dentistry and medicine, infections occur more frequently [7, 8].

Biofilms are particularly a serious challenge in medicine because they firmly adhere to surfaces [9] and can survive in harsh environments [10] by forming a matrix of extracellular polysaccharides [11, 12]. Moreover, they show strong tolerance against chemical and pharmacological therapies [13, 14]. Therefore, it is not only important to remove biofilms for the patient’s health but also to analyze the bacteria involved for more targeted therapies.

Various strategies have been tested to remove biofilms [15–17], and in terms of preserving the viability, low-frequency ultrasound has been identified as the most effective method [18]. Up to 10,000 times more bacteria can be detected that way in the diagnosis of biofilm infections [8, 19].

In dentistry, low-frequency ultrasound is already being used in research to remove and investigate biofilms from surfaces. Since initial microbial colonization takes place within minutes, many studies focus on biofilms due to their constant presence in the oral cavity [20, 21]. Various samples, such as dentures [22], material samples [23], dentin slabs [24], or enamel slabs, have been examined through sonication [25, 26]. The sonicate was used to determine viable bacteria [22–25] or for subsequent PCR analysis [26]. Splint systems are often used to collect in vivo biofilm samples [24–30], since in vitro samples differ significantly [31]. Areas of oral biofilm research include dental material research [24], development of methods [28], new anti-caries strategies [32], characterization of the microbiota on dentures [22], and research on new antimicrobial agents [25, 27].

Ultrasound refers to sound waves with a frequency higher than 16 kHz [8]. In liquids, it can generate microcurrents, shear forces, and cavitation bubbles that detach microorganisms [8, 33, 34]. Ultrasound can be used for sterilization [33] or even induce bacterial growth, depending on the application [35]. Erriu et al. categorized ultrasound based on two main variables: intensity and frequency. Low-frequency ultrasound refers to frequencies below 500 kHz, and low-intensity ultrasound refers to an intensity of less than 3 W/cm^2^ [34].

For the diagnosis of periprosthetic infections, ultrasound with a frequency of 40 kHz and intensity of 0.2–1 W/cm^2^ was used, followed by 30 s of vortexing [8]. This approach can improve the sensitivity of detecting infections in patients, especially after previous antibiotic therapy, and can help to identify low-grade infections in supposedly aseptic prosthesis replacements [6, 8].

No clear recommendation could be found in dental studies regarding the use of low-frequency ultrasound. The duration of ultrasound treatment varied greatly, ranging from 20 s [30] to 20 min [26]. Most studies utilized a simple sonication of 1 to 5 min [22, 24, 25, 27–29, 36, 37], except for one study that used repeated sonication [23]. The frequencies and intensities of ultrasound used were usually not specified. There are limited dental studies investigating the effects of low-frequency ultrasound.

Olsen and Socransky demonstrated that different species of periodontal bacteria react differently to ultrasound, with Gram-positive bacteria being more tolerant than Gram-negative bacteria [38].

In the case of *Actinomyces viscosus* adhered to hydroxyapatite, McInnes et al. [39] found that a sound pressure of at least 25 kPa is required to achieve significant detachment after 60 s. They also observed a consistent two-fold increase in bacterial concentration between 30 s and 6 min of sonication at a constant sound pressure of 50 kPa.

Joyce et al. [40] discovered that using a small sample volume (< 100 ml) and low frequency (38 kHz) and power of 18 W, the bacteria killing rate was higher than the dispersion of the sample after 5 min of sonication.

Wagendorf et al. [41] investigated the use of ultrasound on extracted teeth to better determine the microbial flora of dental infections, which they suggested could potentially result in more precise antibiotic therapy and improved clinical outcomes. They referred to the investigation values for prosthetic infections and utilized a sonication time of 1 min at a frequency of 40 kHz. Similar to studies on prosthetic joint infections, they found a greater diversity through sonication [41].

Overall, the information on sound pressures, frequencies, sonication times, power, or intensities is not consistent, making comparisons difficult. Most studies focus on planktonic specimens or only examine individual species in vitro. However, in vitro samples cannot accurately replicate in vivo biofilm [42].

Because Wagendorf et al. achieved similar improvements in the diagnosis of dental infections as those obtained in studies on prosthetic infections, using the same values for ultrasound, it is reasonable to use their values for frequency and intensity as a reference.

However, since the biofilm structure heavily depends on the species involved [43–46], guideline values should only be provisionally accepted, and further research is needed in the dental field.

The aim of the present study is to propose a standardized sonication time for the dental field to efficiently remove initial oral biofilm while preserving viability. Based on research findings regarding prosthetic infections, a frequency of 35 kHz and an intensity of 0.22–0.87 W/cm^2^ were utilized. The chosen sonication time frame is derived from the most commonly used durations in dental studies. The outcomes of this research can be applied in dental research and the clinical diagnosis of dental infections.

## Material and methods

### Participants

Four healthy volunteers (2 males, 2 females, mean age 25.5 years) were selected for the study after a general anamnesis and thorough examination of their oral health. They had a normal salivary flow rate and buffer capacity and were free of caries as well as any form of periodontal and gingival diseases. Exclusion criteria for the study included pregnancy, smoking, systemic diseases, as well as the use of antibiotics in the last 3 months. Volunteers were informed not to use mouth rinses for 2 weeks prior to and during the study. All volunteers read and signed the informed consent form. The study was approved by the Ethical Committee of the University of Freiburg (No. 91/13).

### Splint system and bovine enamel slabs

**Fig. 1:**
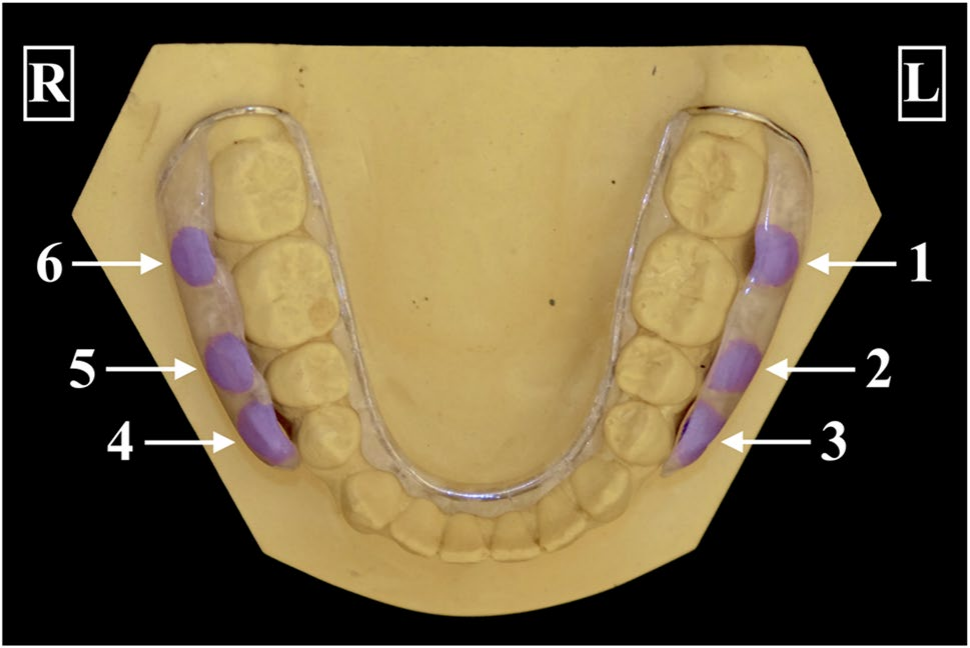
Individual splint for the lower jaw with a buccal acrylic shield that contains six wells to hold bovine enamel slabs, which are facing interdental with their polished surface. Samples in positions 1–5 were sonicated, while the sample in position 6 was not sonicated

Impressions were taken from each participant to fabricate individual splints for the lower jaw. The splints were made using 1 mm CoCr-wire (Wiptam®, Krupp Medizintechnik, Germany) and acrylic resin (Orthocryl®, Dentaurum GmbH, Germany). Each splint had two buccal shields with three wells on each side. These wells were used to hold six cylindrical bovine enamel slabs (BES) on which initial biofilm was formed in vivo (Fig. 1).

The BES were obtained from the incisors of 2-year-old BSE-negative cattle. After extraction, the teeth were cleaned and stored in 0.1% thymol. The samples were prepared using a trephine bur (Komet Dental, Gebr. Brasseler GmbH & Co. KG, Lemgo, Germany) and then ground and polished up to 4000 grit using waterproof silicon carbide paper (Struers GmbH, Ballerup, Denmark) on a wet disc grinder (Knuth-Rotor 3, Struers GmbH, Ballerup, Denmark) as described before [47].

Before insertion into the splint, the BES underwent a cleansing process involving 3 min of ultrasound cleaning with a solution of 3% NaOCl, followed by ultrasound cleaning with 70% ethanol, and finally ultrasound cleaning in double-distilled water for 10 min twice. The cleaned BES were then stored for rehydration in distilled water for 24 h. An addition-cured silicone (Panasil®, Kettenbach GmbH & Co. KG, Germany) was used to insert the BES into the wells of the splint, ensuring that the polished surface of the BES faced the interdental area without touching the gingiva or the surface of the teeth for undisturbed biofilm formation. The BES had a diameter of 5 mm and a height of 1.5 mm (surface 19.63 mm^2^). BES in positions 1–5 were sonicated and used for microbiological analysis of the initial biofilm, while the BES in position 6 was not sonicated and immediately fixed for examination using a high-resolution imaging method.

The participants were instructed to insert the splint at least 2 h after oral hygiene and were advised to place the splint into a cup containing saline solution (0.9% NaCl) while eating or brushing their teeth.

### Study design

The study was divided into three parts (Table 1).

**Table 1:** Overview of the experiments and methodical approaches

Part	Type of sonication	Biofilm samples	Sonication time	Number of samples	Frequency	Intensity	Methods of analysis
1	Simple sonication	4-h matured biofilm	Control (0 min)	20	35 kHz	0.22–0.87 W/cm^2^	- Aerobic and anaerobic CFU (quantification) - MALDI-TOF MS (identification) - SEM-Images (efficiency)
			1 min	20			
			2 min	20			
			4 min	20			
			6 min	20			
2		24-h matured biofilm	Control (0 min)	20			- Aerobic and anaerobic CFU - SEM-Images
			1 min	20			
			2 min	20			
			4 min	20			
			6 min	20			
3	Repeated sonication	24-h matured biofilm	3 × 2 min	20			- Aerobic and anaerobic CFU - SEM-Images

In the first experiment, four participants wore their individual splints for 4 h. To test different sonication times, the participants wore the splint five times. The sonication times were 1 min, 2 min, 4 min, and 6 min, for BES of positions 1–5. BES of position 6 were never sonicated and were immediately fixed for imaging methods. The experiment was performed once without using ultrasound as a control for any sample. The colony-forming units (CFU) were determined aerobically and anaerobically, and the species were identified for the samples from positions 1–5. Afterwards, all BES were fixed for imaging methods. In total, there were 20 biofilm-covered BES available for analyzation for each sonication time and the untreated control.

The second experiment followed a similar procedure, but the participants wore the splints for 24 h. The sonication times were the same as in the first experiment. BES of position 6 served as a control and were fixed for imaging methods. The experiment was also performed once without using ultrasound as a control for any sample. The CFU were determined aerobically and anaerobically for BES of positions 1–5. Afterwards, all remaining BES were fixed for imaging methods to examine if residual adherent biofilms were detectable on the BES after sonication. Overall, there were 20 BES available for analysis for each sonification time and the untreated control.

In the third experiment of the study, the participants wore the splint only once for 24 h. This time the BES of positions 1–5 underwent repeated sonication, with each sample subjected to three consecutive 2-min treatments. After each sonication, the samples were plated to determine the CFU aerobically and anaerobically. Subsequently, the samples were then transferred to new Eppendorf tubes for the next round of sonication, repeating the process three times. Finally, the BES were fixed for imaging methods.

### Ultrasonic treatment

Ultrasonic treatment was used to desorb the microorganisms from the surface of the BES. The BES, covered with an initial biofilm, were removed from the splint using sterile forceps. They were then rinsed in a well plate using 1 ml 0.9% NaCl and transferred into an Eppendorf tube containing 1 ml of reduced transport fluid (RTF) with 25% glucose, as recommended by Syed and Loesche [48]. The tubes were placed in an ultrasonic bath (Sonorex Digiplus DL 255 H, Bandelin, Berlin, Germany) filled with distilled water, ensuring that the water level was at least as high as the surface of the RTF in the Eppendorf tubes. A frequency of 35 kHz and an intensity of 0.22–0.87 W/cm^2^ were used for all sonification times (1 min/2 min/4 min/6 min).

### Colony-forming units (CFU)

After ultrasonication, the samples were vortexed for 30 s at level 9 using a Vortex mixer (Mixer mini Vortex, VWR International bvba, Radnor, USA). A dilution series up to 1:10^4^ was prepared with 0.9% NaCl for each sample. The diluted samples were again vortexed before plating onto Columbia blood agar (CBA; Oxoid GmbH, Wesel, Germany) and yeast-cysteine blood agar (HCB-agar, Institute of Medical Microbiology and Hygiene, Freiburg, Germany). One hundred microliters of the diluted solution was dispensed onto the culture medium using a sterile glass spatula. CBA was used for cultivating aerobic bacteria, which were incubated for 3–5 days in 5% CO_2_ atmosphere and 36 °C. HCB-agar was used for cultivating anaerobic bacteria, which were incubated for 10 days at 36 °C. An anaerobic pot and a gas generator (GENbox anaer, BioMérieux®, Marcy-I Étoile, France) were used to create the proper conditions for anaerobic growth. The number of CFU was determined afterwards for each plate using a colony counter with a magnifying glass (WTW BZG 40, Weilheim, Germany).

### MALDI-TOF MS

The BES (positions 1–5) were separately sonicated, and the samples were then pooled for each sonication time. A diluted series was performed, and the samples were plated on CBA and HCB-agar. The CFU were quantified after 3–5 days of aerobic and 10 days of anaerobic cultivation. All grown single colonies were distinguished based on their color, morphology, and ability to cause hemolysis. The pure bacterial isolates were then analyzed using matrix-assisted laser desorption/ionization time-of-flight mass spectrometry (MALDI-TOF MS) on a MALDI Biotyper® sirius (Bruker, Billerica, MA, USA), following the method described in a previous study [49]. To identify the species, the mass spectra of each bacterial isolate were recorded and compared with a database using the software BioTyper 3.0.

### Scanning electron microscope (SEM)

All biofilm samples before and after sonication were fixed. For this purpose, the BES were stored at 4 °C in 8% formaldehyde for at least 2–3 days. Dehydration was done using an ascending alcohol series of ethanol, followed by drying with increased pressure and temperature using a critical point dryer (CPD 030, BAL-TEC AG, Pfäffikon, Switzerland). Then the BES were sputter-coated using a fine coater (JFC-1200 fine coater, Jeol GmbH, Freising, Germany). After this procedure, all BES were compared using a SEM (JSM-IT100 In TouchScope, Version 1.090, Jeol GmbH, Freising, Germany) to capture images at a magnification of 30–5000-fold at 10 kV.

### Statistical analysis

All computations were based on the log_10_ transformed bacteria concentrations. Median, mean, and standard deviation were calculated for the descriptive analysis of the data. Box plots were created using GraphPad Prism 10.0.1 (GraphPad Software, San Diego, CA, USA) to present the results graphically. Paired *t*-tests were used to compare species and groups among each other and at different sonication times. A mixed linear regression model was used to examine the impact of different sonication times on aerobe and anaerobe bacteria. The determined CFU were used as data basis. The significance level was set at *p* < 0.05. For the exploratory analysis, multiple testing was not corrected. All analyses were performed using Stata 17.0 software (StataCorp, College Station, TX, USA).

## Results

### Single time sonication

**Fig. 2:**
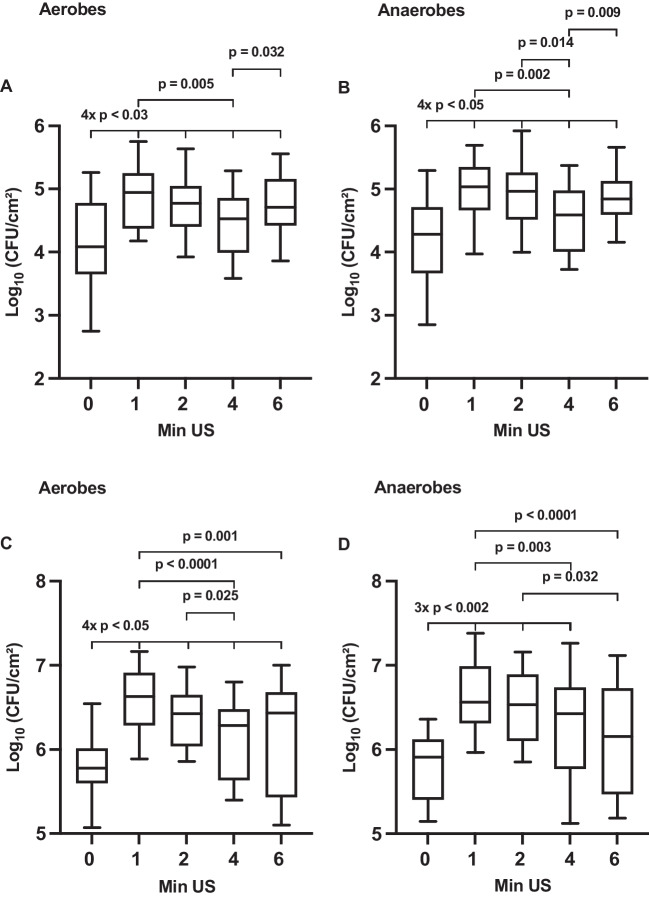
Boxplots showing the impact of various sonication times in minutes (Min US) on the counts of colony-forming units (CFU) per square centimeter for **A** aerobic and **B** anaerobic oral microorganisms of 4-h initial biofilm and for **C** aerobic and **D** anaerobic oral microorganisms of 24-h initial biofilm, all data are presented on a log_10_ scale. The diagram includes the *p*-values obtained from a mixed linear regression model

For an initial biofilm formed for 4 h, all sonication time periods (1 min, 2 min, 4 min, 6 min) resulted in significantly higher counts of CFU for both aerobes and anaerobes compared to the non-sonicated control, as shown in Fig. 2A, B. The highest counts of CFU were observed after 1 min of sonication, representing a 7.2-fold increase for aerobes and a 5.5-fold increase for anaerobes compared to the non-sonicated control. A duration of 4 min showed significantly lower counts of CFU compared to 1 min (aerobes, *p* = 0.005; anaerobes, *p* = 0.002), 6 min (aerobes, *p* = 0.032; anaerobes, *p* = 0.009), and also 2 min of sonication (only for anaerobes, *p* = 0.014). No statistically significant difference was found between 1 min, 2 min, and 6 min of sonication for both aerobes and anaerobes (*p* > 0.05), respectively.

For an initial biofilm formed for 24 h, all sonication time intervals revealed statistically significant higher counts of CFU in aerobes and, with one exception, in anaerobes again compared to the non-sonicated control, as shown in Fig. 2C, D. There was no significant difference in the counts of CFU after 6 min of ultrasonication in anaerobes compared to the non-sonicated control (*p* > 0.05). The highest counts of CFU were again observed after 1 min of sonication, resulting in a 7.1-fold increase for aerobes and a 4.5-fold increase for anaerobes compared to the non-sonicated control. Both 4 min (aerobes, *p* < 0.0001; anaerobes, *p* = 0.003) and 6 min (aerobes, *p* = 0.001; anaerobes, *p* < 0.0001) of sonication showed statistically lower values compared to 1 min. The counts of CFU after 1 min and 2 min of sonication were not significantly different, as well as the counts after 4 min compared to 6 min of sonication in both aerobes and anaerobes (*p* > 0.05).

### Repeated sonication

**Fig. 3:**
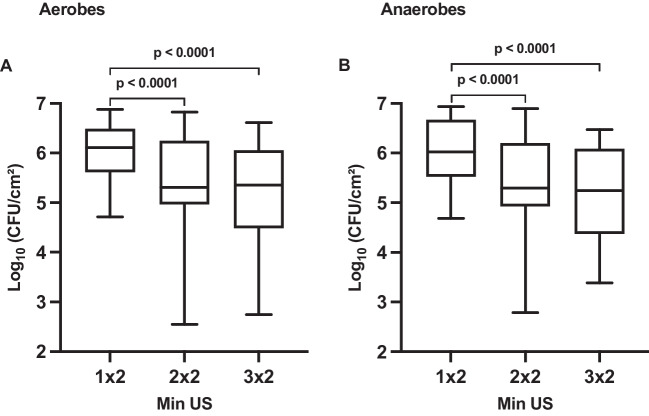
Boxplots showing the impact of repeated sonication of the same BES for 1 × 2 min, 2 × 2 min, and 3 × 2 min (Min US) on the counts of colony-forming units (CFU) per square centimeter for **A** aerobic and **B** anaerobic oral microorganisms of 24-h initial biofilm on a log_10_ scale. The diagram includes the *p*-values obtained from a mixed linear regression model

After the first sonication (1 × 2 min), the highest counts of CFU were reached for aerobes and anaerobes, as shown in Fig. 3. Further CFU were counted after the second (2 × 2 min) and third (3 × 2 min) duration, but with a much wider range of values and statistically significantly lower counts of CFU compared to the first sonication in both aerobes and anaerobes (*p* < 0.0001).

### Microbiological analyses using MALDI-TOF MS

**Fig. 4:**
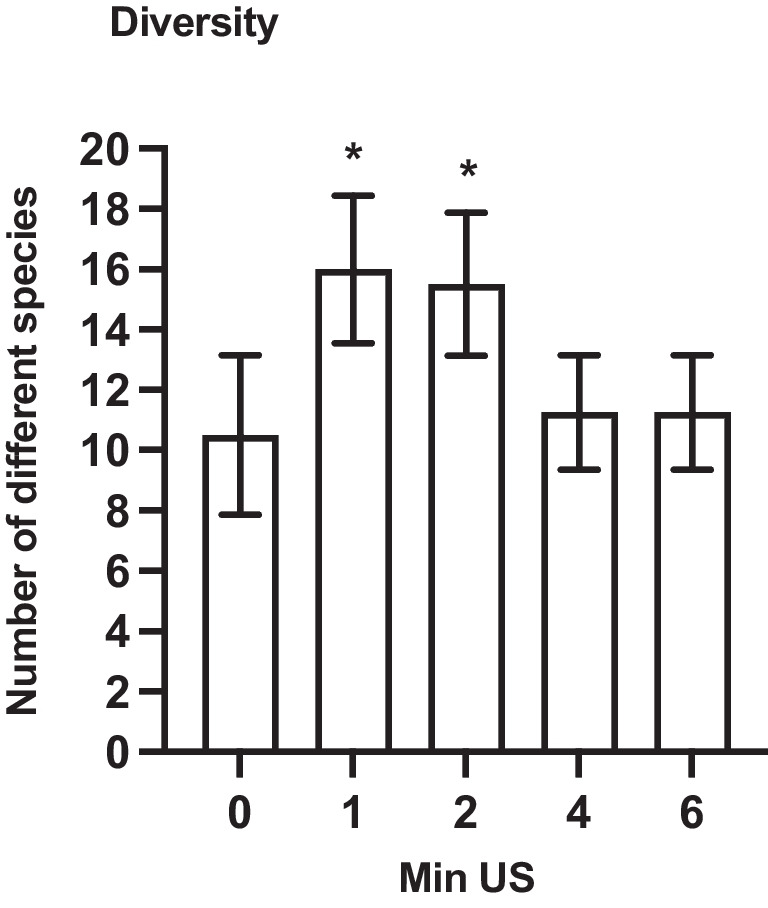
The impact of different sonication times in minutes (Min US) on the bacterial diversity shown as average number of bacterial species of 4-h initial biofilm compared to the non-sonicated control (0 min US). *T*-test: statistical differences (*p* < 0.03) are marked with *

The microbiological analyses showed that the non-sonicated control had the lowest diversity, with a mean value of 10.5 ± 2.29 detected bacterial species, as shown in Fig. 4. The highest diversity was observed after 1 min of sonication, with a mean value of 16 ± 2.12 species. After 1 min and 2 min of sonication, a statistically significant higher bacterial diversity was found compared to not only the non-sonicated control (*p* < 0.03), but also compared to 4 min and 6 min of sonication (*p* < 0.03). There was no statistically significant difference in the bacterial diversity between 1 min and 2 min of sonication, as well as between the non-sonicated control, 4 min, and 6 min of sonication (*p* > 0.05), respectively.

**Fig. 5:**
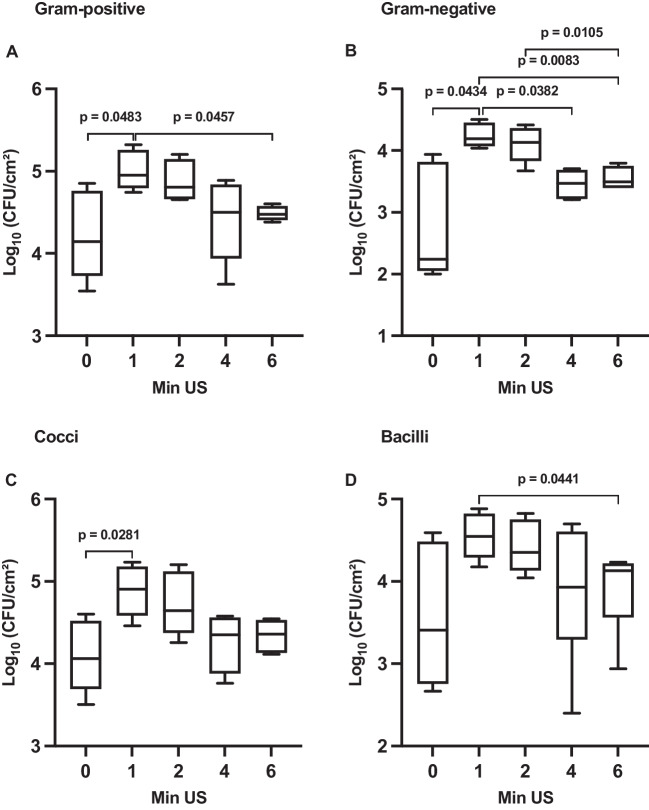
Boxplots showing the impact of various sonication times in minutes (Min US) on the counts of colony-forming units (CFU) per square centimeter for **A** Gram-positive and **B** Gram-negative bacteria and **C** cocci and **D** bacilli of 4-h matured initial biofilm on a log_10_ scale. The diagram includes the *p*-values obtained from a *t*-test

When comparing the counts of CFU at different sonication times, it was found that both Gram-positive and Gram-negative bacteria exhibited the highest counts after 1 min of sonication, as shown in Fig. 5A, B. There was a 4.4-fold increase in Gram-positive bacteria and an 8-fold increase in Gram-negative bacteria compared to the non-sonicated control (Gram-positives, *p* = 0.0483; Gram-negatives, *p* = 0.0434). Additionally, these counts were also significantly higher compared to 6 min (Gram-positives, *p* = 0.0457; Gram-negatives, *p* = 0.0083) and in Gram-negative bacteria also compared to 4 min of sonication (*p* = 0.0382). Furthermore, in Gram-negative bacteria, the counts of CFU were higher after 2 min compared to 6 min of sonication (*p* = 0.0105). However, there was no statistically significant difference observed between 1 min and 2 min of sonication for both Gram-positive and Gram-negative bacteria (*p* > 0.05).

Similarly, cocci and bacilli revealed the highest counts of CFU after 1 min of sonication, as depicted in Fig. 5C, D. There was a 5.5-fold increase in cocci and a 3.5-fold increase in bacilli compared to the non-sonicated control. Statistically significant lower counts of CFU were observed in cocci for the non-sonicated control (*p* = 0.0281) and in bacilli after 6 min of sonication (*p* = 0.0441), in comparison to 1 min.

### Scanning electron microscopic images

**Fig. 6:**
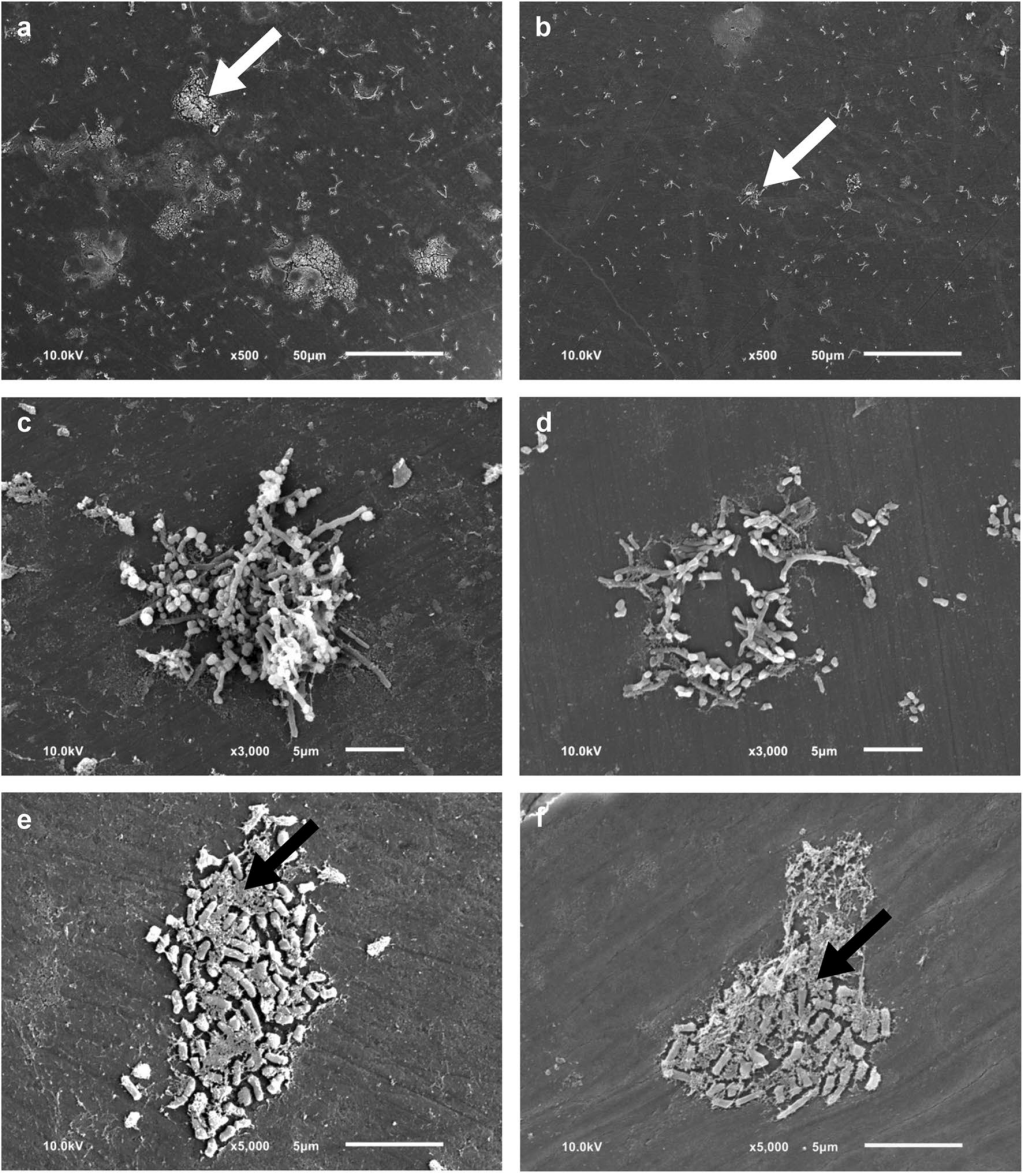
Representative scanning electron microscopic (SEM) images of the surface of BES after a 4-h wearing period are shown at various magnifications (500–5000-fold). Samples of the non-sonicated control (**a**, **c**, **e**) and single-time sonicated samples, from the same participant and wearing cycle (**b**, **d**, **f**). Bacteria (white arrow) appear brightly on the darker enamel surface. Overview image (**a**) after 6 min of sonication (**b**). Intact bacterial colony (**c**) after 2 min of sonication (**d**). Bacteria embedded in the extracellular matrix (black arrow) (**e**) after 6 min of sonication (**f**)

**Fig. 7:**
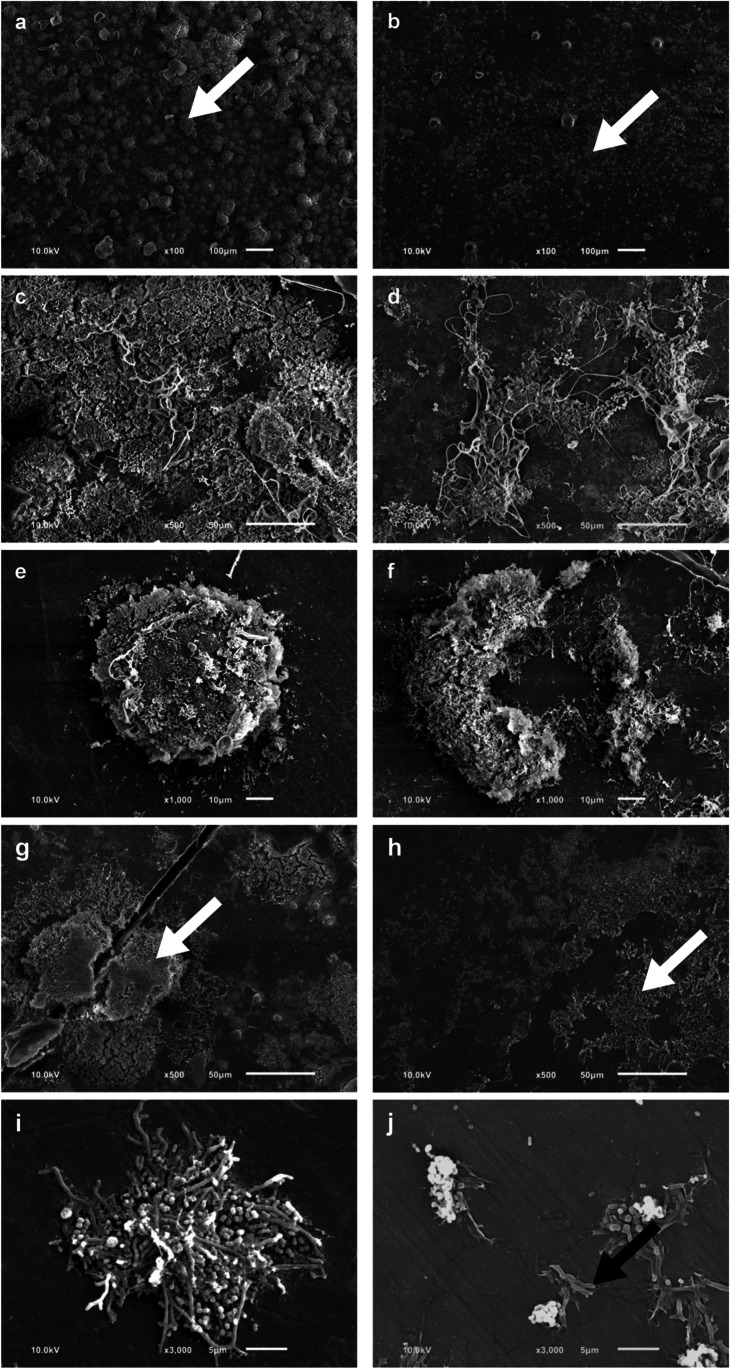
Representative scanning electron microscopic (SEM) images of the surface of BES after a 24-h wearing period are shown at various magnifications (500–3000-fold). Samples of the non-sonicated control (**a**, **c**, **e**, **g**, **i**). Single-sonicated samples (**b**, **d**, **f**) and repeated sonicated sample (**h**, **j**) from the same participant and wearing cycle. Bacteria (white arrow) appear brightly on the darker enamel surface. Overview image (**a**) after 6 min of sonication (**b**). Biofilm formation (**c**). After 1 min of sonication (**d**). Intact bacterial colony (**e**) after 2 min of sonication (**f**). Overview image (**g**) after repeated sonication (3 × 2 min) (**h**). Intact bacteria (**i**). Bacteria with a damaged shape (black arrow) (**j**)

Representative SEM images were selected to illustrate the impact of ultrasonic treatment on 4-h (Fig. 6) and 24-h (Fig. 7) matured initial biofilm at various magnifications. After ultrasonication, the attached biofilm appeared to be reduced compared to the non-sonicated control in samples with both 4-h and 24-h matured biofilms (a, b). The bacteria detached in larger accumulations of three-dimensional colonies (Fig. 6c, d; Fig. 7e, f). In samples with a 4-h matured biofilm, the remaining bacteria were typically directly adhered to the enamel surface as small aggregates (Fig. 6f), while in samples with 24-h matured biofilm, large bacterial colonies were also observed (Fig. 7d, f). It was noticeable that bacteria consistently remained on the surface of the BES even after repeated sonication (Fig. 7g, h) and 6 min of simple sonication (b). Partially, some bacteria were found with a damaged shape after 6 min of repeated sonication (Fig. 7j).

## Discussion

In dental research, low-frequency ultrasound has been widely used to dislodge bacteria from surfaces, facilitating follow-up examinations, i.e., quantification of adherent bacteria and determination of microbial diversity. Currently, research efforts are also being made in the diagnostic field to enhance the diagnosis of microbial composition in the oral cavity using low-frequency ultrasound. This research is based on the application of ultrasound in diagnosing prosthetic joint infections [41]. Unlike in the field of prosthetic infections, there is no standardized recommendation for parameters like sonication time, intensity, or frequency range in the dental field, which makes it challenging to compare results.

The aim of this study was to determine the optimal sonication period for desorbing oral microorganisms embedded in the initial oral in vivo biofilm while preserving their viability as much as possible. Additionally, the present study examined the effectiveness of the different sonication periods for determining the bacterial species present in the initial oral biofilm using the culture technique. To collect test material, an in vivo splint system was used to grow biofilm intraorally from healthy participants. The use of such splint systems to generate in vivo biofilm has already been successfully tested in previous studies [25, 47, 50, 51].

An in vitro study conducted by McInnes et al. [39] revealed that with increasing sonication periods, there is a continuous desorption of microorganisms from the hydroxyapatite surface. The results of our study also demonstrated that not all bacteria were detached at once during repeated sonication, but rather gradually detached with longer sonication times. The study by McInnes et al. suggested that this continuous release of microorganisms leads to a linear increase in the number of CFU. They exhibited a continuous two-fold increase in detected CFU, particularly through dispersion mechanisms [39]. However, *Actinomyces viscosus* a Gram-positive facultative anaerobic rod was the only species tested, and the study was carried out as an in vitro experiment. Our study did not observe a continuous increase in CFU. Instead, we found that the majority of CFUs were counted after 1 and 2 min of ultrasonication, with a significant decrease in CFU counts thereafter. One possible explanation for this discrepancy is the significantly lower frequency of 0.2 kHz used by McInnes et al., which may not lead to such a high bacterial killing rate as the 35 kHz frequency utilized in our study. Furthermore, in our in vivo biofilm samples, we additionally have strict anaerobes that seem to be more sensitive to ultrasound compared to facultatively anaerobic bacteria like *Actinomyces viscosus*. Additionally, Gram-negative bacteria which we observed in our in vivo biofilm samples also appear to be more sensitive to ultrasound than Gram-positive bacteria. Therefore, it can be concluded that *Actinomyces viscosus* may generally exhibit lower sensitivity to ultrasound.

In the in vitro study of Joyce et al. [40], which utilized a similar frequency range as our study, an initial increase in detected CFU of *Bacillus subtilis* was observed, followed by a subsequent decrease, which aligns with the findings of the present study. It was assumed that after 5 min, the killing rate of bacteria outweighed dispersion mechanisms, resulting in a decrease in detected CFU. Based on this, we believe that in our study, the combination of desorption, dispersion, and killing rates contributed to the observed pattern, with the killing rate surpassing further dispersion and desorption of microorganisms already at 4 min of sonication.

However, the study of Joyce et al. was carried out as an in vitro experiment, and there are several possible reasons why the decrease in CFU was observed earlier in our study. Firstly, it is possible that *Bacillus subtilis*, the specific bacterium used in the study of Joyce et al., may be less sensitive to ultrasound compared to the overall bacteria present in the initial biofilm samples tested in the present study. This variation in the sensitivity of different bacterial species to sonication has been observed in other studies [35, 38, 52]. Additionally, the adherence of in vivo oral biofilm to solid surfaces may result in different kinetics influencing the bacteria when exposed to ultrasound compared to the in vitro planktonic samples of single bacterial species, which were used in the study by Joyce et al. [40]. This assumption is supported by the findings of Kobayashi et al. [53], where planktonic in vitro samples and on stainless steel plates adhered in vitro biofilm samples of *S. aureus* were sonicated at 40 kHz. The study revealed significantly fewer counts of CFU in adhered biofilm after only 5 min of sonication, whereas in planktonic samples, it took 30 min of ultrasonication to achieve similar results. Additionally, the PCR results for the detection of adherent bacteria were not dependent on the sonication time, unlike the CFU counts [53]. PCR analysis can determine not only viable bacteria but also dead bacteria, whose proportion presumably increases with longer sonication times [54].

One disadvantage of quantification via CFU is that not all bacteria can be cultured [55], which could further contribute to the discrepancy in results compared to PCR analysis. However, quantifying viable bacteria using CFU made sense in our research project because it is a standard follow-up analysis in dental research to detect initially adherent bacteria [25, 56].

A recent study by Dongre et al. [57], which used ultrasound with a frequency of 40 kHz, concluded that sonication periods longer than 3 min cause cell damage and reduce viability. Overall, our study demonstrates that low-frequency and low-intensity ultrasonication with shorter sonication times of 1–2 min yield better results. This finding is consistent with other studies, including research in the field of diagnosing prosthetic joint infections [8, 40, 53, 57].

Furthermore, by examining the SEM images, we discovered that bacteria consistently remain on the surface even after 6 min of sonication, regardless of whether it was a single or repeated sonication process. These findings have also been observed in other studies [34, 39, 58]. In samples taken after 4 h, the remaining bacteria were primarily in the form of rods, while in samples from biofilms that were formed in vivo for 24 h, various types of bacteria and larger areas of remaining biofilm were observed. In samples with 24-h old biofilm, it was more common to find blank areas in between the biofilm where whole bacterial aggregates seemed to have been torn out by sonication. The dense network of polysaccharides formed after 24 h of bacterial formation may have contributed to better adhesion and cohesion of the bacterial aggregates, potentially providing enhanced protection. It is also suggested that an increased cell concentration and a greater abundance of matrix may have increased the viscosity of the fluid, subsequently reducing the forces of cavitation due to changes in fluid properties [39, 53]. Consequently, it is not possible to definitively determine the total bacterial diversity and number of adherent bacteria for either 24-h or 4-h matured biofilms through follow-up analyses. It must be acknowledged that certain bacterial species may be over- or under-represented.

A previous study by Hannig et al. [59] using transmission electron microscopy has shown that the efficiency of removing pellicle layers from enamel surfaces can be increased by combining 30-min ultrasonication with additional methods. For instance, when ultrasonication was combined with calcium chloride, complete removal of the outer globular pellicle layer was achieved [59]. Although the paper focused on removing pellicle instead of detaching bacteria and preserving their viability, combining ultrasonication with other methods is an aspect to be explored in future studies to further optimize the efficiency of biofilm removal.

Using the culture technique and identifying the isolated bacteria using MALDI-TOF MS, we found that the diversity was highest with a 1.5-fold higher number of detected species after 1 min of sonication compared to the non-sonicated sample.

A previous study has shown that oral microorganisms in the initial salivary pellicle adhere as aggregates to the enamel surface [60]. The application of ultrasound can lead not only to the desorption of microbial aggregates but also to the dissolution of various microbial species within such aggregates. By using fluorescence in situ hybridization, we were indeed able to show that the aggregates of the initial oral adhesion are composed of several different bacterial species [47]. Since a colony-forming unit can include one microbial species or many different taxa, the ultrasonication may cause the dispersion of aggregates and a better distribution on the agar plates That may lead to more detected CFU of different bacterial species and therefore a higher detected bacterial diversity. This highlights the role of ultrasound treatment in the analysis of initial biofilm.

As in our study, other studies have also reported a significantly higher diversity after a sonication time of 1 min [41, 61]. Portillo et al. [61] conducted a study comparing sonication fluid cultures of in vivo samples of prosthetic components and tissue cultures. The prosthetic components were sonicated for 1 min at a frequency of 40 ± 5 kHz. They found that sonication resulted in the detection of 30% more pathogens compared to non-sonicated tissue culture. Wagendorf et al. [41] used similar sonication values in their study. They compared sonication with swabs and vortexing of in vivo samples of removed teeth and found a significantly higher diversity in the sonicated samples. Both studies utilized in vivo samples and similar sonication values as the present study. However, it is important to note that both studies only compared methods and did not investigate different sonication periods. Therefore, there are no in vivo studies available with similar sonication values that compare the effects of varying sonication times on biofilms.

The subsequent decrease in diversity after 4 min of ultrasonication as observed in the present study could be attributed to the varying sensitivity of bacterial species to ultrasound [33, 38, 52].

The results of the present study revealed that Gram-negative bacteria were more sensitive to ultrasonication compared to Gram-positive bacteria, which is consistent with the results of previous studies [33, 38, 52].

Monsen et al. [52] evaluated the in vitro effects of ultrasound with a frequency of 40 kHz performed on six planktonic bacterial species and also found Gram-negative species like *Haemphilus influenzae*, *Pseudomonas aeruginosa*, and *Escherichia coli* to be more effected by ultrasound than Gram-positive bacteria like staphylococci and streptococci.They suggested the dispersion of aggregates and chains might be the reason for Gram-positive species being less effected by ultrasound, because Gram-negative bacteria normally do not form aggregates during planktonic culture. However, the interaction of bacteria in biofilms is significantly more complex, and properties such as forming aggregates in planktonic samples plays a less important role in the analysis of in vivo biofilm and therefore do not seem to be the only reason for this effect.

Olsen and Socransky [38] compared the ultrasonic sensitivity of 14 planktonic bacterial species associated with periodontal diseases in an in vitro study. Gram-negative organisms appeared again less resistant to sonication than Gram-positives and can be divided into groups according to their relative sensitivity, showing that there are relatively less sensitive Gram-negative species such as *Eikenella corrodens* and very sensitive genera such as *Bacteroides* or *Wolinella*. They suggest that these groups form due to differences in cell wall structure. *E*. *corrodens* has a distinct peptidoglycan layer, which is absent in *Wolinella* and may lead to lower sensitivity to ultrasound in *E. corrodens* [38]. Scherba et al. [54] hypothesized that ultrasound may impact the inner cytoplasmic membrane, which could explain why not only Gram-negative bacteria, but also species of Gram-positive bacteria which lack an outer membrane exhibit sensitivity to sonication.

Additionally, in the present study, cocci were found to be less sensitive to ultrasound compared to bacilli or rods, which has also been reported previously [38, 60]. Olsen and Socransky suggested that bacteria had to be aggregated to be less exposed to the forces of ultrasound, so that the sensitivity might reflect the ability of the species to form aggregates with actively motile rods like *Wolinella* be the most sensitive organisms to ultrasound [38]. Another possible explanation for this observation is that rods have a larger surface area exposed to ultrasound [62]. The structural damage observed in rod-shaped bacteria in the SEM images further supports the notion that they are particularly affected by ultrasound.

The increased sensitivity of anaerobic bacteria compared to aerobic bacteria aligns with findings in research on prosthetic joint infections [8, 17]. This could be attributed to the generation of excess oxygen through ultrasound-induced cavitation [40, 41].

Since the present study does not aim to investigate any epidemiological parameters such as microbial diversity, but to provide a proof of concept to the effect of sonication time on studying the initial oral biofilm, the small number of four participants was sufficient to generate the in situ biofilm samples.

The fluctuations shown in the present study have been also observed in previous studies as well [26, 63]. Nonetheless, future studies should consider including a larger number of subjects to account for the high inter- and intra-individual fluctuations. For instance, when investigating the potential diagnostic application of dental infections, it would be beneficial to include risk groups or individuals with illnesses rather than solely healthy subjects, as this would be the focus of improved diagnostics. Additionally, the study should encourage further exploration of both the frequency and ultrasound intensity to examine their effects on bacteria and desorption of the in vivo formed oral biofilm, with the aim of establishing standardized recommendations that can be utilized in research and diagnostics within the dental field.

## Conclusions

Overall, our study demonstrated that when using low-frequency, low-intensity ultrasound to detach bacteria from surfaces, shorter sonication times, particularly 1 min and 2 min, resulted in higher bacterial counts and greater diversity. Additionally, we observed that some bacteria consistently remain on the surface. The results of the present study suggest that the outcome of clinical studies using oral in situ biofilm models and sonication is influenced by the interaction of desorption, dispersion, and killing rate factors. Furthermore, it was evident that the impact of ultrasound on bacteria varies depending on the species. Anaerobes, bacilli, and Gram-negative bacteria appear to be more sensitive to ultrasound, indicating that a sonication time of 1 min would be a conservative approach for all species. Moreover, the treatment of biofilm-covered material surfaces with low-frequency ultrasound, regardless of the microbiological analysis used ex vivo, does not appear to be sufficient enough for harvesting the biofilm microbiota. A DNA extraction for subsequent microbiome analysis using next-generation sequencing would be more sufficient if it would be conducted directly on the adherent biofilms, rather than on the biofilm samples obtained through the use of low-frequency ultrasound. These findings should be taken into consideration in future clinical studies.
